# Transcriptional regulation of lipid metabolism by fatty acids: a key determinant of pancreatic β-cell function

**DOI:** 10.1186/1743-7075-2-1

**Published:** 2005-01-05

**Authors:** Zahra Fatehi-Hassanabad, Catherine B Chan

**Affiliations:** 1 Department of Biomedical Sciences, University of Prince Edward Island, 550 University Avenue, Charlottetown, PE C1A 4P3 Canada

## Abstract

**Background:**

Optimal pancreatic β-cell function is essential for the regulation of glucose homeostasis in both humans and animals and its impairment leads to the development of diabetes. Type 2 diabetes is a polygenic disease aggravated by environmental factors such as low physical activity or a hypercaloric high-fat diet.

**Results:**

Free fatty acids represent an important factor linking excess fat mass to type 2 diabetes. Several studies have shown that chronically elevated free fatty acids have a negative effect on β-cell function leading to elevated insulin secretion basally but with an impaired response to glucose. The transcription factors PPARα, PPARγ and SREBP-1c respond to changing fat concentrations in tissues, thereby coordinating the genomic response to altered metabolic conditions to promote either fat storage or catabolism. These transcription factors have been identified in β-cells and it appears that each may exert influence on β-cell function in health and disease.

**Conclusion:**

The role of the PPARs and SREBP-1c as potential mediators of lipotoxicity is an emerging area of interest.

## Introduction

**Figure 1:**
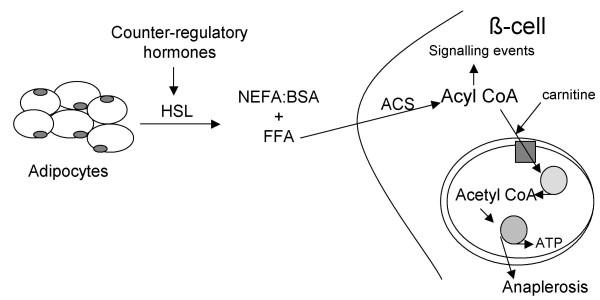
**Schematic diagram of fatty acid metabolism in the fasted state**. Counter-regulatory hormones such as catecholamines act on adipocytes to increase lipolysis via hormone-sensitive lipase (HSL). Circulating FFA enter the cell and are converted to acyl CoAs, catalyzed by acyl CoA synthase (ACS). Acyl CoA enter the mitochondria via carnitine palmitoyl transferase-I (solid square) and enter the β-oxidation cycle (stippled circle) to produce acetyl CoA that is then available for further metabolism in the TCA cycle, leading to increased ATP and substrates for anaplerosis. In the β-cell, acyl CoA also participate as signalling molecules to promote insulin secretion (see text).

Fatty acids are physiologically important both structurally, as components of phospholipids and glycolipids, as well as functionally, as fuel molecules. Metabolites of fatty acids, such as leukotrienes or prostaglandins, act as potent mediators in many biological processes. Fatty acids provide energy [1,2], particularly in the fasted state (Figure 1), but abnormalities in the metabolism of fatty acids can contribute to the pathogenesis of obesity and type 2 diabetes.

## Type 2 diabetes and free fatty acids

Diabetes affects 6 % of the adult population and, with a growth rate of 6% per year, it is estimated that 200 to 300 million people worldwide will be afflicted by the end of this decade [3]. Type 1 diabetes, which accounts for < 10 % of all cases of diabetes [4], results from autoimmune-mediated destruction of pancreatic β-cells. The destruction may occur over months to years and can result in complete loss of the endogenous insulin supply and therefore results in exogenous insulin dependency.

Type 2 diabetes, which accounts for 90 to 95 % of diabetes cases worldwide, is a heterogeneous disorder and its prevalence is rising. Type 2 diabetes is accompanied by chronic insulin resistance and a progressive decline in β-cell function [5]. Obesity is a major risk factor for the development of type 2 diabetes [6] and is believed to confer increased risk through obesity-associated insulin resistance [7]. Type 2 diabetes is often associated with hypertriglyceridemia or increased circulating concentrations of free fatty acids (FFA) [8]. Therefore, type 2 diabetes can be considered a lipid disorder as well as a disease of glucose intolerance [9].

## Metabolism of fatty acids in the beta cell and insulin secretion

Fatty acids, not glucose, are the major endogenous energy source for unstimulated islets [10]. This is consistent with the observation that although islets contain little glycogen, they maintain high rates of oxygen consumption in the absence of glucose [11]. Stimulation of islets by glucose diminishes fatty acid oxidation and increases total respiration [12]. Thus, rising post-prandial plasma glucose shifts the β-cells from fatty acids to glucose as an oxidative fuel. However, plasma concentrations of other nutrients such as FFA and amino acids can modulate the process of glucose-induced insulin secretion [9]. The plasma levels of nutrient metabolites vary with dietary composition. Thus, feeding behavior plays an important role in the control of islet β-cell function [13,14].

Short-term (2–6 hours) elevation of the plasma FFA concentration in human subjects [15] and animals [16,17] enhances while an acute decrease inhibits glucose-stimulated insulin secretion [15,18]. Following lipid infusion or ingestion of a mixed meal, the plasma FFA concentration rises and FFA diffuse into the β-cells [19]. Within the cytosol, fatty acids are converted to their fatty acyl CoA derivatives (Figure 1), which in turn augment insulin secretion via different signalling mechanisms: increased formation of phosphatidic acid and diacylglycerol, which directly and indirectly (through activation of protein kinase C) enhance exocytosis of insulin stored within secretory granules; stimulation of endoplasmic reticulum Ca^2+^-adenosine triphosphatase, leading to an increase in intracellular calcium concentration and augmentation of insulin secretion; and closure of the K^+^- ATP channel with resultant depolarization of the β-cell membrane, which causes an increase in intracellular Ca^2+ ^and stimulation of exocytosis of insulin-containing granules [21]. In addition to being oxidized, glucose can be metabolized through anaplerotic processes to increase malonyl CoA concentrations in the β-cell. Malonyl CoA inhibits CPT-I, thus impairing the transport of fatty acyl CoAs into the mitochondria where they would be oxidized [20,21]. The fact that *de novo *fatty acid synthesis in the β-cell is very low [22] indicates that malonyl-CoA is used as a switch compound, not as a precursor or effector molecule like long chain fatty acyl-CoA. The cytosolic concentration of long chain fatty acyl-CoA is controlled by feedback inhibition of acyl-CoA synthetase, and is buffered by fatty acid and long chain fatty acyl-CoA binding proteins [23]. The total concentration of long chain fatty acyl-CoA in livers of fed and fasted rats, is about 95 and 220 nmol/g dry weight, respectively [24], however quantification of cytosolic long chain fatty acyl-CoA in other tissues has yet to be done.

In contrast to the acute effect of elevated plasma FFA to enhance insulin secretion, longer-term (> 48 h) exposure results in an impaired β-cell response to glucose both *in vitro *and *in vivo *in animals [25,26] and humans [27-31]. The inhibitory effect of chronically elevated plasma FFA is more prominent in individuals with a genetic predisposition to develop type 2 diabetes [32], thus a reduction in the plasma FFA concentration in type 2 diabetes improves insulin secretion [32,33]. The term lipotoxicity describes the deleterious effect of chronic FFA elevation on insulin secretion from the pancreatic β-cell [34]. In the Zucker diabetic fatty rat, chronically increased plasma FFA levels lead initially to a physiological impairment in insulin secretion. With time, β-cell mass is reduced by more than 50 % [26]. Within the β-cell, elevated fatty acyl CoAs increase the formation of ceramide, a sphingolipid. Ceramide, in turn, augments the formation of the inducible isoform of nitric oxide, which is toxic to the β-cell [35]. Incubation of human islets with FFA or ceramide has been shown to cause β-cell apoptosis [36].

## Transcriptional regulation of free fatty acid metabolism

Free fatty acid metabolism responds to varying metabolic states partially by induction of enzymes that promote either catabolic or anabolic processes. There are two major classes of transcriptional regulators of enzymes involved in fatty acid metabolism, the peroxisome proliferator-activated receptors (PPARs) and the sterol regulatory element binding proteins (SREBPs), which both exist in several isoforms. In general, PPARγ and SREBP-1c regulate processes involved in lipogenesis whereas lipolytic enzymes are induced by PPARα [37].

## Peroxisome proliferator-activated receptors

The PPARs form a subfamily in the nuclear receptor superfamily. PPARs, like other nuclear receptors, regulate gene expression in response to specific ligands through their actions as transcription factors. Peroxisomes contain PPAR-regulated enzymes involved in fatty acid β-oxidation [38]. Genetic deficiencies in peroxisome biogenesis in the human cause an accumulation of long chain fatty acids in cells [39]. So far, three isoforms encoded by separate genes and designated PPARα, PPARδ and PPARγ have been identified [40].

### PPARα

PPARα was the first member of this nuclear receptor subclass to be described. PPARα is expressed in numerous metabolically active tissues including liver, kidney, heart, skeletal muscle, brown fat [41-43], and also in monocytes [44], vascular endothelium [45] and vascular smooth muscle cells [46].

**Figure 2:**
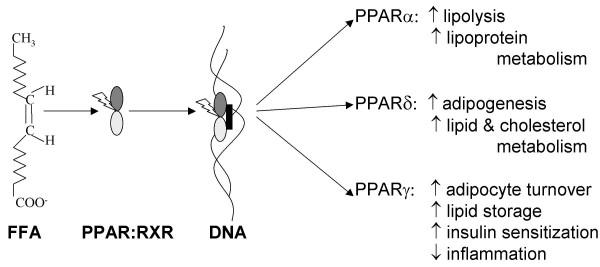
**Overview of PPAR activation and effects**. FFA (eg. oleic acid) interact with PPAR, which dimerize with retinoid X receptor (RXR) and translocate to the nucleus where the complex interacts with PPRE to activate gene transcription. The general effects of transcriptional activation of PPARα, PPARδ and PPARγ are shown on the right of the figure.

PPARα plays an important role in the regulation of cellular uptake, activation and β-oxidation of fatty acids. The natural, preferentially-binding ligands of PPARα are long chain unsaturated fatty acids including arachidonic acid, linoleic acid, and oleic acid but saturated fatty acids like palmitic acid can also act as ligands [47]. In hepatocytes and other tissues where it has been studied, ligand-activated PPARα binds to peroxisome proliferator response elements (PPRE) of DNA (Figure 2) and up-regulates transcription of genes involved in lipid catabolism and lipoprotein metabolism (Table 1) [48,49]. Consequently PPARα serves as a long chain fatty acid sensor that leads to autoregulation of long chain fatty acid metabolism mainly in the liver and heart and to a lesser extent in muscle, thus decreasing tissue content of lipids and minimizing lipotoxicity as circulating levels fluctuate [50]. Activation of PPARα also induces hepatic proliferation, hepatomegaly and hepatocarcinogensis in animal [51] but not human liver [52]. Obesity is a major risk factor in the development of type 2 diabetes and PPARα may affect body weight through regulation of fatty acid catabolism or expending energy [53]. PPARα ligands (such as fibrate drugs) could therefore improve insulin sensitivity by reducing lipid accumulation in tissues [54].

**Table 1:** Selected hepatic PPARα regulated genes with at least one functional peroxisome proliferator receptor element (PPRE) identified within the promoter sequence

**Gene**	**Function**	**Species**	**References**
Acyl CoA binding protein	fatty acyl-CoA ester transport	rat	127
Acyl CoA oxidase	peroxisomal β-oxidation	rat, human	128-130
Apolipoprotein-AI and AII	plasma HDL metabolism	human, mouse, rat	131-134
Apolipoprotein-AV	plasma triglyceride metabolism	human	134
Apolipoprotein-CIII	plasma HDL metabolism	rat	135
Bifunctional enzyme	peroxisomal β-oxidation	rat	136
Carnitine palmitoyl transferase-I and -II	mitochondrial β-oxidation	human, mouse, rat, hamster	132, 137-139
Cytochrome P450 enzymes	fatty acid and cholesterol metabolism	rat, mouse, human	130, 141-145
Δ6- and Δ5-desaturase	desaturation of fatty acyl-CoA	mouse	146
Fatty acid binding protein	fatty acid binding/transport	mouse	147
Fatty acid transport protein and translocase	fatty acid transport	mouse	148, 149
Lipoprotein lipase	triglyceride clearance	mouse	148, 149
Liver X receptor α	cholesterol metabolism	mouse	150, 151
Long-chain acyl-CoA synthetase	fatty acid activation	human, mouse	139, 152
Malic enzyme	fatty acid synthesis	mouse, rat	153, 154
Mitochondrial HMG-CoA synthase	ketogenesis	rat, human	152, 155
Medium-chain acyl-CoA dehydrogenase	mitochondrial β-oxidation	mouse	138, 139
Phospholipid transfer protein	HDL metabolism	human	156
Stearoyl-CoA desaturase-1	desaturation of fatty acyl CoA	mouse	157
Superoxide dismutase	free radical metabolism	rat	158
Thiolase B	mitochondrial β-oxidation	rat	159
Transferrin	iron transport	human	160
Very long- and long-chain acyl CoA dehydrogenases	mitochondrial β-oxidation	mouse	139

**Abbreviations: **HDL, high density lipoprotein; HMG-CoA, hydroxymethylglutaryl-Coenzyme A

### PPARδ

PPARδ was initially reported as PPARβ in *Xenopus laevis *[49]. Subsequently, the receptor was cloned in the human [55] as well as in rodents [56] and was named PPARδ. PPARδ is expressed in a wide range of tissues and cells with the highest levels of expression found in digestive tract, heart, kidney, liver, adipose and brain [57]. Saturated and unsaturated fatty acids are natural ligands for PPARδ [58,59]. PPARδ is implicated in adipocyte differentiation, which is induced by long-chain fatty acids [60]. In skeletal muscle, activation of PPARδ results in induction of proteins involved in lipid catabolism, cholesterol efflux and respiratory coupling in skeletal muscle independent from the effects of PPARα and PPARγ agonists [61].

### PPARγ

PPARγ stimulates fatty acid storage in adipose tissue by increasing both the storage capacity and the fatty acid flux into adipocytes. PPARγ is expressed in many cell types, including epithelial cells, B and T cells, macrophages, endothelial cells, smooth muscle cells [62,63] and predominantly in adipose tissue where it is necessary for the differentiation of adipocytes [64]. There are 2 splice variant of the isoform called PPARγ1 and γ2; the expression distribution of PPARγ2 is more limited than that of PPARγ1 [65].

The natural ligands of PPARγ are several unsaturated fatty acids such as oleate, linoleate, eicosapentaenoic and arachidonic acids [53]. Members of the thiazolidinedione (TZD) family, which are known as antidiabetic compounds, are synthetic ligands of PPARγ [54]. In adipocytes, PPARγ increases the expression of numerous genes involved in lipid metabolism and uptake [66,67]. Activation of PPARγ also induces adipocyte apoptosis, which is restricted primarily to large fully differentiated adipocytes [68]. This pro-apoptotic effect of PPARγ activation on large adipocytes, coupled with its capacity to enhance differentiation of adipocytes *de novo*, favours the formation of small adipocytes that tend to replace the large adipocytes normally constituting white adipose tissue [68].

PPARγ also negatively regulates transcription of several genes that impair insulin action, including tumor necrosis factor-α (TNFα) and leptin, proinflammatory cytokines produced by adipocytes and associated with insulin resistance [69-72]. Thus, the TZD drugs lower hyperglycemia, hyperinsulinemia and hypertriglyceridemia by indirectly enhancing the sensitivity of tissues to insulin, especially in skeletal muscle. However, the function of PPARγ is not restricted to adipogenesis and insulin sensitization. In peripheral monocytes and macrophages, PPARγ agonists inhibit the production of inflammatory cytokines [73] and induce differentiation and apoptosis in various cancer cells [74,75].

## Peroxisome proliferator-activated receptors and β-cell function

Both PPARα and PPARγ have been detected in pancreatic β-cells [76,77]. One caveat that complicates interpretation of some of the work described below is that PPARα and PPARγ agonists have effects on β-cell function independent of their interaction with the transcription factors. Thus, both fibrates and TZD can alter ATP-dependent K channel activity and rapidly (within 10 minutes) increase insulin secretion [78]. In addition, the generalized metabolic effects of these compounds may mean that effects observed *in vitro *on isolated islets may not apply to the *in vivo *situation. Therefore, the mode of delivery of the agents (directly onto islets *versus *in diets) and the time frame of study are important considerations.

In pancreatic islets, exposure to long chain fatty acids (mixed unsaturated and saturated) induces PPARα expression [76] whereas high glucose *in vitro *or hyperglycemia *in vivo *suppresses expression [79,80]. Artificial ligands of PPARα such as WY14643 and clofibrate also induce PPARα expression in rat islets [76,81,82]. Similar to hepatocytes, this leads to up-regulation of enzymes favouring lipolysis, including acyl-CoA oxidase [76,81], pyruvate dehydrogenase-4 [82] and CPT-I [76,81].

The question arises as to the role of PPARα in the physiological regulation of insulin secretion. Its induction by long chain fatty acids and its ability to augment the insulin response to low glucose [81] suggests that it may play a role in sustaining β-cell secretory capacity during normal, cyclical periods of fasting. Thus, when glucose is low, PPARα will be induced, favouring β-oxidation of lipids to maintain β-cell ATP at a maintenance level. Moreover, the ability of β-cells to oxidize lipids is a critical for resumption of glucose-stimulated insulin secretion at the end of the fasting period [17]. However, when glucose is elevated above basal, PPARα will be reduced, allowing efficient glucose metabolism-dependent insulin secretion while inhibiting fatty acid oxidation. Overall, the effect of oscillating PPARα activity inversely with glucose concentration may help to maintain glucose responsiveness of the β-cell [83]. Four-to-six-hour fasted PPARα KO mice had normal circulating insulin [84,85] and their islets had normal glucose sensitivity [84] whereas 24 hour fasted mice had a 3-fold increase in circulating insulin [85]. The longer-term fast would allow for greater adaptation to occur; higher fasting insulin may reflect hepatic insulin resistance rather than altered β-cell function.

In addition to these postulated mechanisms of PPARα control over β-cell glucose and lipid metabolism, it has also been proposed that amino acid metabolism might be affected. In the liver, an increase in PPARα is associated with a decrease in amino acid catabolism [86]. Because glutamine metabolites are potential signaling molecules in the β-cell [87], PPARα induction under conditions of low glucose could impair glucose-stimulated insulin secretion via its effects on glutamine catabolism [83]. This hypothesis has yet to be proven.

In pathophysiological conditions involving deranged glucose and lipid metabolism, altered expression of PPARα may be important in the β-cell's lack of glucose responsiveness. In Zucker diabetic fatty rat islets, despite chronic hyperlipidemia, expression of PPARα, acyl-CoA oxidase and CPT-I mRNA is reduced [88]. It has thus been proposed that glucose is the dominant regulator of PPARα in the β-cell and that its suppression is a component of glucolipoxicity [89].

Glucolipotoxicity is a state in which β-cells are exposed to elevated plasma concentrations of both glucose and FFA, as is the case in insulin resistance. Several signalling pathways of the β-cell may be affected by altered PPARα expression and the overall outcome is predicted to depend upon whether fat or glucose has the dominant effect. In cases where glucose is elevated relative to lipid, a chronic reduction in PPARα would be expected to decrease the lipid oxidizing capacity of the β-cell [88,89], eliminating a detoxification route for fat metabolites [89]. Accumulation of lipids, for example as triglyceride within the β-cell, is associated with impaired glucose-stimulated insulin secretion, increased ceramide formation and apoptosis [88]. When lipid is chronically elevated relative to carbohydrate, induction of PPARα presumably would cause strong up-regulation of fat oxidizing genes but also UCP2 (see below), which would suppress glucose-stimulated insulin secretion. The implication of these hypotheses is that either too much or too little PPARα would impair β-cell function. Evidence in the literature supports this contention when *in vitro *models are employed. Notably, culture of islets or INS-1 cells with high glucose (6–20 mM) for 48 hours strongly suppresses PPARα protein expression by 80%. As predicted, fatty acid oxidation and glucose-stimulated insulin secretion are attenuated, while islet triglyceride and lipid esterification are increased [79]. Conversely, induction of endogenous β-cell PPARα (with clofibrate) leads to an increase in CPT-I expression and fatty acid oxidation, resulting in blunted basal and glucose-stimulated insulin secretion [90]. However, the situation is less clear when experiments are performed *in vivo*, leading to the conclusion that activation of PPARα in tissues other than β-cells causes indirect effects on insulin secretion secondary to changes in peripheral insulin sensitivity [83]. Thus, type 2 diabetic mice given dietary WY14,643, a PPARα agonist, have normalized serum lipids, glucose and insulin. PPARα activation also improves glucose-stimulated insulin secretion, reduces β-cell proliferation and β-cell mass compared with untreated controls [91]. Similarly, fenofibrate-treated obese diabetes-prone OLETF rats retain β-cell mass and have lower islet triglyceride content and fatty oxidation than untreated animals [92]. In both cases, the effects on β-cells are likely secondary to the observed weight loss and increase in insulin sensitivity of peripheral tissues.

Chronic induction of PPARα may influence also insulin secretion indirectly because PPRE have been found in the promoter region of uncoupling protein-2 (UCP2) [93]. In general, uncoupling proteins (numbered 1–3 in order of their discovery) decrease metabolic efficiency by dissociating ATP synthesis from substrate oxidation in the mitochondrion by promoting translocation of protons from the inter-membrane space, across the inner mitochondrial membrane to the matrix [94]. Therefore, circumstances that limit mitochondrial proton gradient formation, such as up-regulation of UCP2 expression and activity, are predicted to limit insulin secretion. A study specifically examining the role of PPARα by use of the ligand clofibrate demonstrated induction UCP2 in islets [90]. In liver, stimulation of PPARα (or PPARδ when PPARα was absent) caused induction of UCP2 [95]. UCP2 expression inversely correlates with β-cell ATP and glucose-stimulated insulin secretion [96-99]. The significance of these findings is that up-regulation of UCP2 expression suppresses glucose-stimulated insulin secretion and is implicated as a potential contributor to lipotoxic effects mediated by PPARα in β-cells.

PPARγ may also be an important transcriptional regulator of both normal and abnormal metabolism in pancreatic β-cells. In hyperglycemic, pancreatectomized rats the expression of PPARγ mRNA is increased [80] but others found that high fat but not high glucose up-regulates PPARγ protein expression *in vitro *[100]. In adipocytes, PPARγ alters the expression of fat metabolizing enzymes to increase FFA uptake into storage while simultaneously preventing the release of FFA [66,67]. However, in the β-cell some actions of PPARγ seem to mimic those of PPARα. Thus, one of the earliest demonstrations in islets of direct activation of PPARγ showed that TZD caused mobilization of triglyceride and increased FFA oxidation in Zucker diabetic fatty rats [101], resulting in improved insulin secretion [101,102]. This observation has been reinforced in more recent work. Induction of PPARγ by three different methods enhances expression of genes that participate in fatty acid oxidation [103]. Glucose-stimulated insulin secretion is enhanced by both PPARα and -γ agonists in *db/db *mice [104]. Consistent with this, mice with a partial global knockdown of PPARγ (PPARγ^+/-^) on a high fat diet had blunted glucose-stimulated insulin secretion in isolated islets that was associated with an islet-specific accumulation of triglyceride [105] even though insulin resistance was partially prevented [106].

The TZD increase glucokinase and GLUT2 expression and activity via interaction with PPRE in the respective gene promoters [107,108]. A PPARα-agonist also induced GLUT2 expression in islets but the effect on glucokinase was not documented [109]. Improved glucose metabolism, however, has not been a consistent outcome of PPARγ induction [103]. Nonetheless, overexpression of PPARγ in a β-cell line is detrimental to glucose-stimulated insulin secretion and proinsulin synthesis, with PPARγ agonists causing a further negative effect [110]. Since PPARγ was not detected in control cells, it is unclear whether these results are physiologically relevant to primary β-cell function. Interestingly, in rodent islets PPARα is expressed at higher levels than PPARγ [76], while in human islets the situation is reversed [111]; the functions regulated by PPARα in rodents may be more pertinent to PPARγ in human β-cells.

PPARγ activation also regulates some β-cell functions that have not been ascribed to PPARα. PPARγ activation by TZD may relieve oxidative stress in β-cells of diabetic animals [112], leading to preservation of β-cell mass [104,112-114] and partial improvement in glucose-stimulated insulin secretion from isolated islets [112]. The potential anti-oxidative or anti-inflammatory effects of TZD in islets of type 2 diabetes models are interesting in light of reports that TZD reduce diabetes incidence in non-obese diabetic (NOD) mice [115] and more generalized inflammatory/immune responses in a variety of tissues [116]. Moreover, PPARγ appears to be a critical determinant of β-cell expansion in response to a high fat diet [117]. However, despite these studies showing that PPARγ exists in β-cells and that its activation can regulate gene expression and cell function, Rosen et al. [117] recently showed that the TZD's antidiabetic effects are still fully present in mice in which PPARγ has been specifically eliminated only in β-cells. Thus, the dominant effects of dietary TZD on insulin secretion are likely indirect, a consequence of improved lipemia and glycemia.

## Sterol regulatory element binding protein

The family of SREBPs governs transcriptional activation of a large number of genes involved in regulation of lipid metabolism, including lipogenesis, cholesterol transport and synthesis [118]. Of interest is the high expression of SREBP-1c in liver and adipose tissue [119], and its detection in pancreatic β-cells [120]. The primary function of SREBP-1c is to regulate transcription of genes involved in lipogenesis, such as acetyl-CoA carboxylase, fatty acid synthase and steroyl-CoA desaturase [119] and enzymes of glycolysis [118,119]. In the liver SREBP-1c appears to mediate the transcription of most insulin-responsive genes and in turn its expression, and possibly its activation, are induced by insulin [119]. Thus, SREBP-1c activity is enhanced during periods of dietary plenty; when glucose is abundant and insulin is stimulated. The outcome of SREBP-1c activation is to promote fat-sparing, leading to an increased synthesis of saturated and monounsaturated fatty acids, triglycerides and phospholipids, as well as enhanced glucose utilization via the glycolytic pathway [119]. Elevation of SREBP-1c in obesity characterized by hyperinsulinemia may therefore explain the onset of fatty liver.

SREBP-1c appears to have a similar function in lipogenesis in pancreatic β-cells as in hepatocytes, but the effects on glycolytic enzymes have received little attention. Notably, blockade of SREBP-1c expression attenuates the glucose-induced increase in acetyl-CoA carboxylase activity seen in control β-cells [121] whereas an increase in SREBP-1c induces lipogenic enzymes, triglyceride accumulation and UCP2 expression [105,122-124]. The outcome of elevated SREBP-1c is a decrease in glucose metabolism and glucose-stimulated insulin secretion in all cases. Consistent with these studies utilizing molecular manipulation of SREBP-1c expression, studies of Zucker diabetic fatty rats demonstrate increased SREBP-1c levels in islets [120]. SREBP-1c has also been implicated as a regulator of apoptosis in β-cells [122]; thus the loss of β-cell mass seen in obese-diabetic models might be related to events triggered by this transcription factor. Indeed, β-cell apoptosis might be under control of both PPARγ and SREBP-1c because TZD has been reported to block the increase in SREBP-1c in diabetic fatty rats [120]; this implies that PPARγ regulates SREBP-1c. Conversely, other groups have evidence that SREBP-1c can up-regulate PPARγ mRNA expression [103,123] ; thus, the relationship between these two factors is not yet clear. The UCP2 promoter has a sterol response element [124] so the negative effects of SREBP-1c on insulin secretion might be caused by its induction of UCP2. However, reducing UCP2 expression by means of a small interfering RNA only partially restored glucose-stimulated insulin secretion in SREBP-1c-overexpressing cells. Likewise, activation of the AMP-activated kinase partially rescued the phenotype of the cells with SREBP-1c induction [125]. Certainly, SREBP-1c is implicated as a key contributor to lipotoxicity, as proposed elsewhere [89,126] but further research is required to fully understand its role in regulating insulin secretion in health and diabetes.

## Conclusions

FFA exert dual effects on insulin secretion, dependent on the duration of exposure. Acute exposure to FFA increase glucose-stimulated insulin secretion whereas chronic exposure attenuate glucose sensitivity of pancreatic β-cells. The coordinated control of these processes by lipid-sensing transcription factors and its relevance to β-cell dysfunction in type 2 diabetes mellitus is increasingly a subject of investigation.

PPARs (especially PPARα and PPARγ) are involved in the long-term regulation of lipid metabolism and their activity is modulated by endogenous lipid-derived ligands. PPAR agonists have positive effects on glucose homeostasis and lipid metabolism and can reduce cardiovascular events in obese-diabetic patients. PPARα is a fasting lipid oxidation-glucose sparing regulator whereas PPARγ is post-prandial lipid storing-glucose utilizing regulator. In islets, however, both PPARα and -γ appear to have some functions more consistent with PPARα, particularly induction of lipid oxidizing enzymes, which is potentially particularly important for maintaining basal insulin secretion. Growing evidence suggests that PPARγ is a regulator of β-cell proliferation and that PPARγ agonist-mediated anti-oxidative effects may also contribute to anti-diabetic activity.

SREBP-1c up-regulates lipogenic enzymes in β-cells as it does in liver. Its chronic induction in islets of obese-diabetic rodents may therefore contribute to lipotoxicity by promoting triglyceride accumulation and removing fatty-acid derived signalling factors from the cellular pool. SREBP-1c and PPAR functions appear to be closely linked through cross-talk between the pathways that control their own expression, and may function in concert to affect not only fatty acid metabolism but also glucose metabolism, β-cell proliferation and apoptosis.

Drugs given orally to activate PPARs can improve insulin sensitivity of peripheral tissues and generally appear to enhance β-cell function secondary to their insulin-sensitizing effects. However, it remains possible that specific effects on β-cells are also important contributors to the positive metabolic effects of PPAR agonists in type 2 diabetes treatment.

## Declaration of Competing Interests

The author(s) declare that they have no competing interests.

## Authors Contributions

ZF-H and CBC contributed equally to the writing of this review.
